# Exploring desert-adapted beetles with 3D geometric morphometrics

**DOI:** 10.1038/s41598-025-05967-1

**Published:** 2025-07-02

**Authors:** Tomasz Szara, Marcin Jan Kamiński, Ozan Gündemir

**Affiliations:** 1 Department of Morphological Sciences, Institute of Veterinary Medicine, Warsaw University of Life Sciences-SGGW, 02-776 Warsaw, Poland; 2 Zoological Museum, Museum and Institute of Zoology, Polish Academy of Sciences, Twarda 51/55, 00-818 Warszawa, Poland; 3 Department of Anatomy, Faculty of Veterinary Medicine, Istanbul University-Cerrahpasa, 34320 Istanbul, Turkey

**Keywords:** Ecology, Zoology

## Abstract

Darkling beetles (Tenebrionidae) are a diverse and ecologically significant group, particularly in arid environments, yet their taxonomy remains problematic due to scarcity of phylogenetic contributions and robust morphological investigations. This study applies 3D geometric morphometrics to evaluate the taxonomic validity and morphological differentiation among three closely related sandworm beetle taxa: *Gonopus deplanatus*, *G. tibialis kalaharicus*, and *G. tibialis punctatus*. Seventy-nine museum-preserved specimens were digitized using a 3D scanner, with multiple scans taken from six different orientations to ensure comprehensive surface reconstruction. Twenty-one anatomical landmarks were assigned to the prothorax (12 landmarks) and pterothorax (9 landmarks), targeting significant taxonomic features like pronotal width, elytral curvature, and the structure of the prosternal process. Based on this dataset, the size and shape variation were analyzed. Results suggest that *G. t. punctatus* displayed a narrower pronotum and more pronounced prosternal process than *G. t. kalaharicus* and *G. deplanatus*, indicating a previously underappreciated taxonomic distinction. For elytra, the differences among taxa were primarily observed in width, curvature, and declivity. Additionally, the stronger correlation between pterothorax shape and size suggests that larger individuals tend to have more pronounced and rounded elytra, potentially influencing thermoregulation or structural reinforcement in response to environmental pressures. These results show the transformative potential of 3D geometric morphometrics in entomological systematics by providing a quantitative framework for distinguishing closely related beetle taxa. These findings suggest that traditional taxonomic criteria may have underestimated the extent of variation between these taxa, underscoring the importance of integrating advanced morphometric approaches into systematic studies. Furthermore, detecting allometric influences in shape variation provides new insights into potential functional and ecological adaptations, which may be linked to the distinct desert environments these beetles inhabit.

**Supplementary Information:**

The online version contains supplementary material available at 10.1038/s41598-025-05967-1.

## Introduction

Darkling beetles (Tenebrionidae Latreille) form the primary faunal component of numerous arid and semiarid ecosystems worldwide^1–15^and in specific arid habitats, they play a vital role in element circulation^16^. The omnipresent xerophily of this phylogenetic lineage has fascinated researchers from various fields. As such, some of the darkling beetle species have been incorporated into biology-inspired engineering projects as models for seeking solutions for efficient cooling and hydration systems^17,18^. Nevertheless, due to the largely outdated and confusing taxonomy, the potential of most Tenebrionidae remains unexplored. This tendency seems especially undesirable in light of the ongoing desertification of many terrestrial ecosystems^19^.

**Fig. 1:**
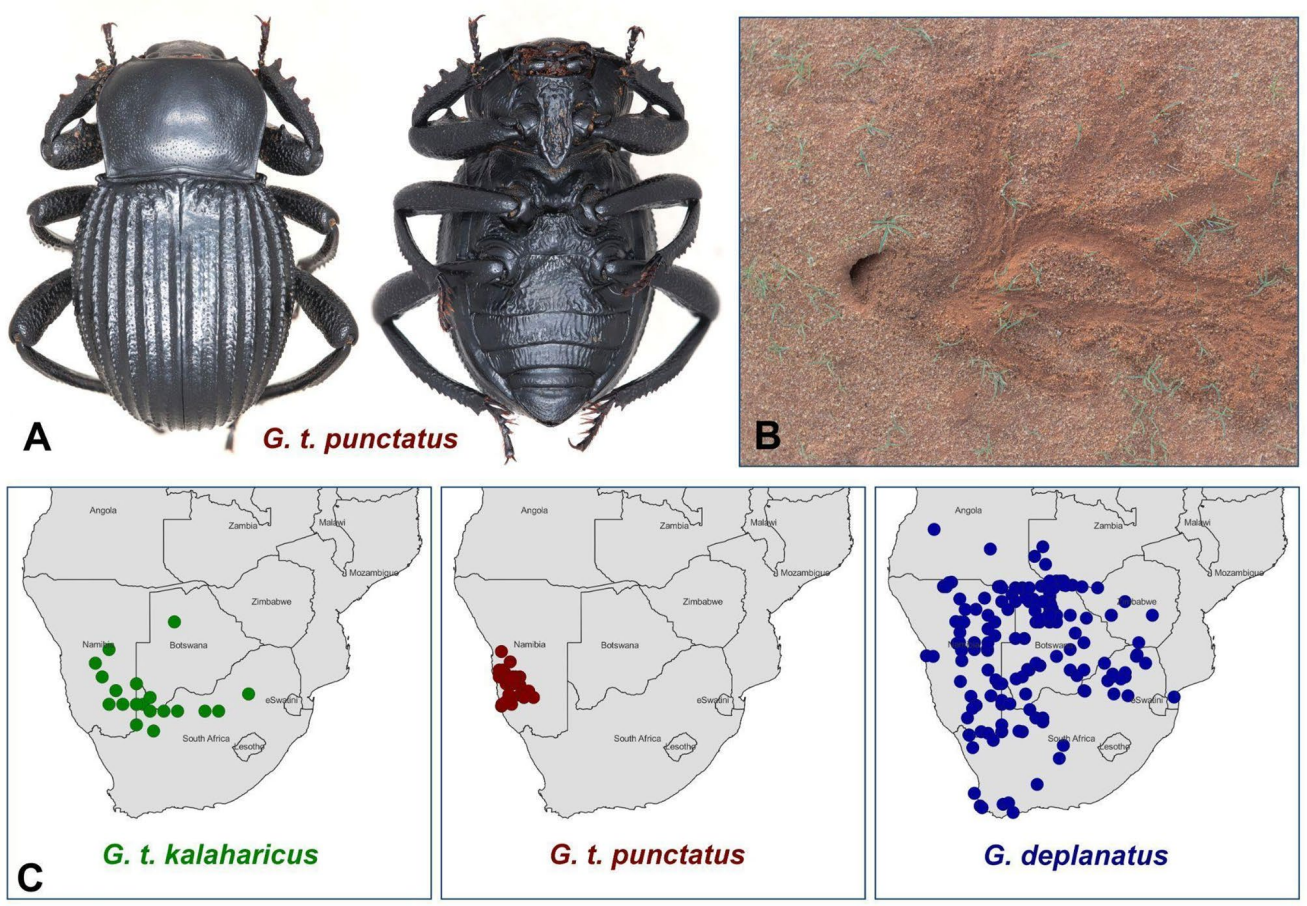
Morphology, habitat, and distribution of sandworm beetles. (A) Holotype of *Gonopus tibialis punctatus* (dorsal and ventral view). (B) Burrow entrance and sand-trail pattern of *Gonopus deplanatus* (photo by Christopher Charles Wirth). (C) Distribution of the three studied taxa based on Endrödy-Younga^20^.

Sandworm beetles (Fig. 1A), constituting the genus *Gonopus* Latreille, comprise 21 species and subspecies divided over two subgenera – *Agonopus* Gebien and *Gonopus*^20,21^. Members of this group, ranging in size from 13 to 24 mm, are relatively large and represent one of the most prevalent insect lineages in many South African deserts^1,22^. They play a crucial role in those ecosystems, serving as food for other organisms and spatially transforming these environments^22^. The adult sandworm beetles are nocturnal fossorial detritivores that shelter in self-made burrows during the daytime (Fig. 1B). Those tunnels often cohabit with different groups of arthropods, which helps them to survive in the harsh desert environment^23,24^. *Gonopus* (*Gonopus*) *deplanatus* Fåhraeus and *G. (Gonopus) tibialis* Fabricius constitute one of the most commonly collected species of sandworm beetles in the Namib and Kalahari deserts^20^(MJK pers. obs.).

Apart from the nominal form, six subspecies have been recognised within *G. tibialis*^20^. The status of some of them remains highly questionable due to their sympatric distributions. Furthermore, the diagnostic features of many of those taxa are extremely subtle, precluding clear delimitations^20^. A good example constitutes *G. tibialis kalaharicus* Endrödy-Younga (Kalahari endemic) and *G. tibialis punctatus* Endrödy-Younga (Namib desert endemic) (Fig. 1C), which are mainly distinguished based on the presence/absence of tubercle row on the last elytral interval and pattern differences in punctuation of the pronotal disc – features proven to be variable across the whole genus. Despite the excellent availability of entomological materials concerning sandworm beetles in the World’s Natural History Museums (MJK pers. obs.), no morphometric studies were ever conducted to test the validity of currently designated species and subspecies, leaving the potential of quantitative features fully unexplored^20^.

Traditional linear morphometric methods have been widely implemented in entomological research, focusing mainly on wing morphology^25^. The advantage of this method over the conventional approach was proven in different aspects, from evolutionary research^26,27^ to taxonomy^28^. Nevertheless, this method suffered from its limitations. For instance, it fails to fully capture the complex three-dimensional shape variation in the morphology of different groups of animals. This limitation is particularly evident in taxa with subtle structural differences, where traditional measurements may overlook key diagnostic traits^29,30^. As a response, 3D geometric morphometrics, which entirely omits such restrictions, has become the leading method for investigating size and shape variation in the anatomical structures of organisms^31^. The advantages of this methodology have already been proven for many disciplines, especially in osteology^32,33^. However, the applications of this approach in entomology remain untested. This can be potentially driven by the small size of many insect groups, which might preclude data collection using standard methodologies. Nevertheless, due to the exoskeleton structure that provides easily definable landmarks, arthropods constitute a promising group of animals that might highly benefit from the recent development of 3D geometric morphometrics.

This study employs 3D geometric morphometrics to assess the morphological variation among closely related taxa of desert-adapted sandworm beetles (*Gonopus deplanatus*, *Gonopus tibialis kalaharicus*, and *G. t. punctatus*), to determine whether shape differences provide robust evidence for taxonomic separation. Beyond taxonomic validation, this study explores the relationship between morphology and ecological adaptation. Since these taxa inhabit different desert environments, analyzing their prothorax and pterothorax morphology can offer insights into potential functional adaptations related to locomotion, burrowing behavior, or environmental pressures. Additionally, by implementing landmark-based 3D shape analysis, this research aims to establish a replicable methodological framework for future studies, encouraging the broader application of geometric morphometrics in insect systematics, evolutionary biology, and ecological morphology.

## Materials and methods

### Insects

Two closely related subspecies of sandworm beetles, *Gonopus* (*Gonopus*) *tibialis kalaharicus* and *Gonopus* (*Gonopus*) *tibialis punctatus*, were selected for the study. Furthermore, the more distantly affiliated and morphologically distinct species^20^*Gonopus* (*Gonopus*) *deplanatus*, has been implemented in the analyses to test the reliability of the applied methodology to harvest taxonomically important quantitative data. A total of 79 pinned, museum-preserved specimens were examined. This included 20 individuals of *G. t. kalaharicus*, 24 of *G. t. punctatus*, and 35 of *G. deplanatus*. Label data and repositories for the studied specimens are presented in the Appendix S1. Taxonomic identifications were made by using Endrödy-Younga^20^ and by referring to type materials. Morphological terminology follows that of Doyen & Tschinkel^34^.

### Specimen digitization and landmarking

**Fig. 2:**
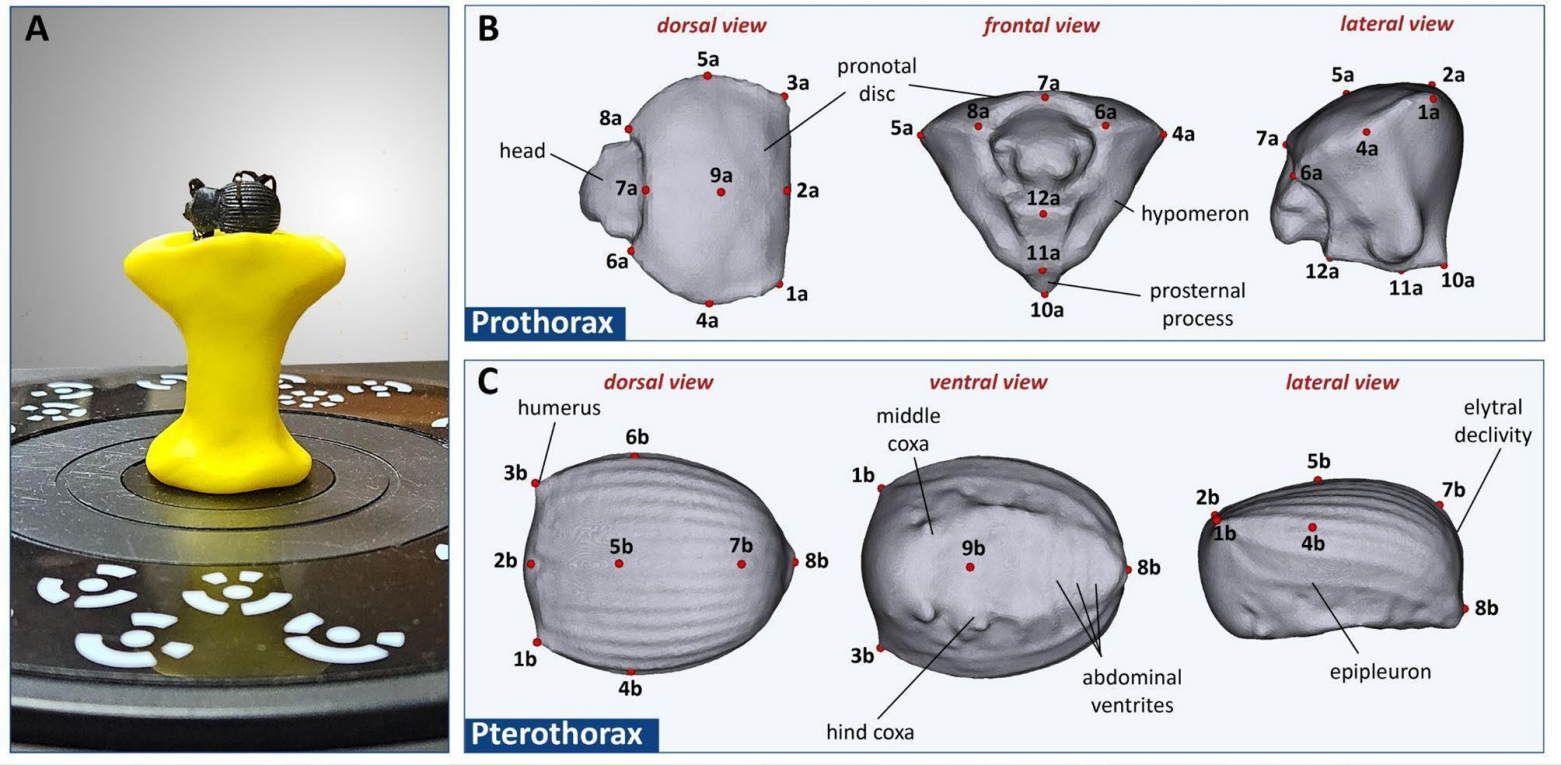
Overview of study design. (A) Scanning process of a *Gonopus deplanatus* specimen. (B) Twelve prothoracic landmarks positioned on the pronotal disc and prosternal process. (C) Nine pterothoracic landmarks distributed across the elytra and abdominal ventrite I. See Table 1 for detailed landmark descriptions.

Entomological labels were taken off, and the pinned beetles were mounted onto a clay platform (Fig. 2A). To acquire full 3D models, each specimen was scanned in different orientations to ensure complete surface reconstruction. The scanning process was performed in six positions: left side, right side, dorsal (elytra), ventral, elytral tip, and an additional angled position to capture complex structures. A Shining 3D EinScan Pro 2 × 3D scanner (Shining 3D, Hangzhou, China) was used to create high-resolution 3D models of the studied beetle specimens. Scanning was conducted using a Shining 3D rotary table to enhance accuracy and minimize occlusions. EXScan Pro (v4.0.0.4), provided by the scanner manufacturer (Shining 3D, Hangzhou, China), was used for 3D model reconstruction, ensuring high-detail preservation of morphological features despite the challenges posed by the relatively small size (13–24 mm) and delicate structures of the beetles (e.g., antennae, legs).

**Table 1:** Description of landmarks used in geometric morphometric analysis of the studied sandworm beetles.

Functional tagma	Area	Landmark no	Description
Prothorax	Basal pronotal angles	1a, 3a	Corresponding to left and right angles
	Middle of the pronotal base	2a	Landmark was positioned in the middle of the pronotal base, above the scutellum.
	Maximum pronotal width	4a, 5a	Two landmarks were positioned on opposite lateral edges of the pronotal disc at the widest point.
	Anterior pronotal angles	6a, 8a	Corresponding to left and right angles
	Middle of apical pronotal edge	7a	The landmark was positioned in the middle of the pronotal apical edge
	Center of the pronotal disc	9a	Landmark situated at the intersection of lines connecting 2a-7a and 4a-5a
	Tip of the prosternal process	10a	Located at the tip of the prosternal process, before its declivity
	Intercoxal point (procoxa)	11a	Situated between the procoxae
	Apex of the prosternal process	12a	Tip point of the pronotal collar, situated in line with 10a and 11a
Pterothorax	Elytral humeri	1b, 3b	Corresponding to the left and right humeri
	Scutellum	2b	Located in the middle of the elytral base, on the basal edge of the scutellum
	Maximum elytral width	4b, 6b	Two landmarks were positioned on opposite lateral edges of the elytra at the widest point.
	Middle of the elytral disc	5b	Landmark situated at the intersection of lines connecting 2b-8a and 4b-6a
	Elytral declivity	7b	Located on the elytral suture at declivity (level of base of 4th abdominal ventrite)
	Elytral tip	8b	Situated on elytral suture
	Intercoxal point (hind coxa)	9b	Situated between the hind coxae

A total of 21 anatomical landmarks corresponding to commonly used taxonomic traits, such as maximum width of pronotum and elytra, were defined on the exoskeleton of studied beetles (Table 1; Fig. 2). To avoid artifacts caused by functional mobility of given body parts, the landmarks were subdivided over and analyzed exclusively within two subgroups – prothorax (landmarks on pronotal disc and prosternal process) and pterothorax (landmarks on elytra and first abdominal ventrite). Given the common bauplan of beetle anatomy, the implemented landmarks are easily replicable and can be applied even in the case of distantly related taxa^35^. 3D Slicer software (version 5.2.2) was used for landmarking. Initially, a draft set of landmarks was created, which was then consistently applied to all samples by the same researcher to ensure accuracy in digitization^36^. All 21 landmarks were successfully used on every studied individual.

### Data analysis

Landmark configurations were processed using Generalized Procrustes Analysis (GPA) to eliminate non-shape variations such as size, position, and orientation^4,41^. Principal Component Analysis (PCA) was then applied to explore the primary axes of shape variation, retaining only principal components (PCs) that accounted for more than 10% of the total variation for further analyses^31^.

To assess patterns of shape variation and morphological differences among groups, we performed a Procrustes ANOVA on the aligned landmark configurations. This analysis evaluated whether species exhibited significant differences in their mean shapes. A permutation-based approach with 1,000 iterations was used to determine statistical significance. Additionally, pairwise Procrustes ANOVA comparisons were conducted to assess morphological differences between species further, quantifying the magnitude and statistical significance of shape variation among specific groups. All analyses used the geomorph package (v.4.0.9) in R (RStudio 2024.09.1)^37^.

Size variation was analyzed based on centroid size (CS) to assess overall differences in specimen size independently of shape data. This metric represents the absolute size of individuals and was used to determine allometric effects^31^. A one-way ANOVA (Analysis of Variance) was performed on the centroid size data to test for significant differences in size among groups. To identify which groups differed from each other, Tukey’s HSD (Honestly Significant Difference) test was applied as a post-hoc analysis. Additionally, to assess the effect of allometry, a multivariate linear regression analysis was conducted to examine the relationship between centroid size and the first three principal components (PC1, PC2, and PC3) obtained from PCA.

## Results

### Shape and size variation

Procrustes ANOVA revealed significant variation in prothoracic shape among the three studied taxa (*G. deplanatus*, *G. t. kalaharicus*, *G. t. punctatus*), with an R² of 0.221, F = 10.752, and *P* = 0.001. This R² value indicates that approximately 22% of the total shape variation is attributable to differences among taxa (Tables 2 and 3). Pairwise analyses showed significant prothoracic shape variation in the following combinations: *G. deplanatus* and *G. t. kalaharicus* (R² = 0.127, F = 7.734, *P* = 0.001), *G. deplanatus* and *G. t. punctatus* (R² = 0.182, F = 12.708, *P* = 0.001), and *G. t. kalaharicus* and *G. t. punctatus* (R² = 0.221, F = 11.885, *P* = 0.001).

**Table 2:** Comparative assessment of inter-species variation in shape and size for the prothorax.

Response variable	Predictor variable	SS	MS	*R*²	F	Z	*P*
Shape	All taxa	0.14456	0.072278	0.22055	10.7520	8.0325	0.001
Shape	*G. deplanatus-G. t. kalaharicus*	0.074956	0.074956	0.1273	7.7336	5.3252	0.001
Shape	*G. deplanatus-G. t. punctatus*	0.11365	0.11365	0.1823	12.7083	6.1050	0.001
Shape	*G. kalaharicus-G. t. punctatus*	0.013455	0.013455	0.2206	11.8847	5.9057	0.001
Size	All taxa	1.140e-30	5.700e-31	0.4377	29.5782	5.2582	0.001
Size	*G. deplanatus-G. t. kalaharicus*	9.310e-31	9.310e-31	0.4422	42.0110	4.3583	0.001
Size	*G. deplanatus-G. t. punctatus*	1.213e-30	1.213e-30	0.3752	34.2229	3.9716	0.001
Size	*G. t. kalaharicus-G. t. punctatus*	3.25e-31	3.25e-31	0.0210	0.8990	0.4378	0.350

**Fig. 3:**
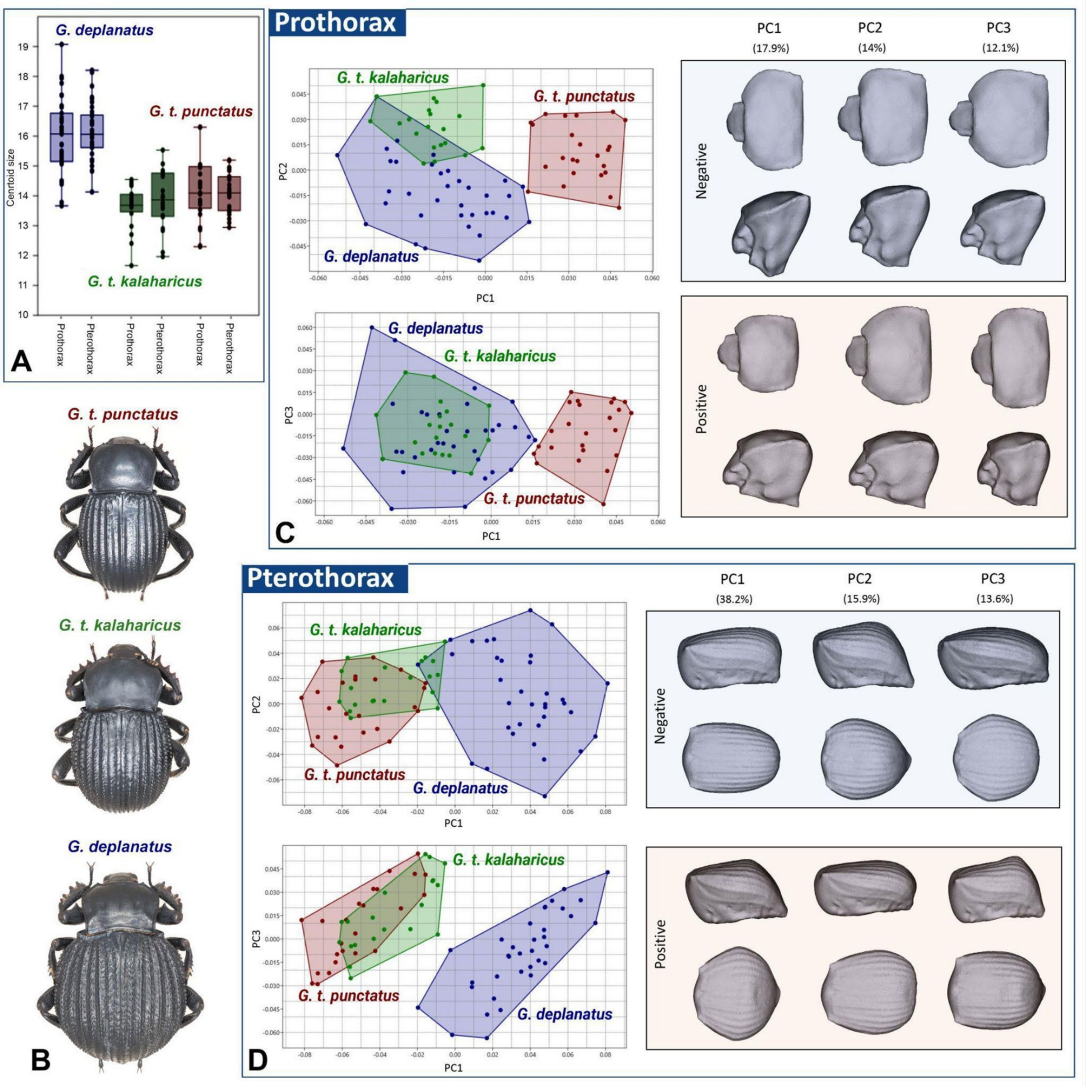
Size and shape variation in studied sandworm beetles (*G. deplanatus*,* G. t. kalaharicus*,* G. t. punctatus*). (A) Centroid size differences across taxa for the prothorax and pterothorax. (B) Habitus morphology of the three taxa. (C) PCA results for the prothorax, with models showing shape variation along PC1, PC2, and PC3. (D) PCA scatter plots for the pterothorax with corresponding shape models.

Prothoracic size also varied significantly among the taxa (R² = 0.438, F = 29.578, *P* = 0.001), with size differences accounting for approximately 43.8% of the total variation (Tables 2 and 3). Pairwise results indicated significant size variation between *G. deplanatus* and *G. t. kalaharicus* (R² = 0.442, F = 42.011, *P* = 0.001) and between *G. deplanatus* and *G. t. punctatus* (R² = 0.375, F = 34.223, *P* = 0.001). However, the analysis between *G. t. kalaharicus* and *G. t. punctatus* showed no significant size variation (R² = 0.021, F = 0.899, *P* = 0.350) (Fig. 3A).

**Table 3:** Comparative assessment of inter-species variation in shape and size for pterothorax.

Response variable	Predictor variable	SS	MS	*R*²	F	Z	*P*
Shape	All taxa	0.07106	0.035529	0.34835	20.3140	6.9031	0.001
Shape	*G. deplanatus-G. t. kalaharicus*	0.025751	0.0257508	0.27861	20.4700	5.8418	0.001
Shape	*G. deplanatus-G. t. punctatus*	0.044687	0.044687	0.34530	30.0630	5.5154	0.001
Shape	*G. kalaharicus-G. t. punctatus*	0.03547	0.035470	0.09298	4.3053	3.2111	0.002
Size	All taxa	1.170e-31	5.873e-32	0.67646	79.4520	8.1717	0.001
Size	*G. deplanatus-G. t. kalaharicus*	6.560e-31	6.562e-31	0.67665	110.9068	5.9376	0.001
Size	*G. deplanatus-G. t. punctatus*	4.000e-32	3.987e-32	0.60169	86.1058	5.3107	0.001
Size	*G. t. kalaharicus-G. t. punctatus*	3.40e-31	3.396e-31	0.11607	5.5151	1.9671	0.021

For the pterothorax, Procrustes ANOVA identified significant shape variation among the taxa (R² = 0.348, F = 20.314, *P* = 0.001), with shape differences explaining 34.8% of the total variation (Table 3). Pairwise analyses revealed significant shape variation between *G. t. kalaharicus* and *G. t. punctatus* (R² = 0.093, F = 4.305, *P* = 0.002), *G. t. kalaharicus* and *G. deplanatus* (R² = 0.279, F = 20.470, *P* = 0.001), and *G. t. punctatus* and *G. deplanatus* (R² = 0.345, F = 30.063, *P* = 0.001). Pterothoracic size also showed significant variation among taxa (R² = 0.676, F = 79.452, *P* = 0.001), accounting for 67.6% of the total variation (Table 3). Pairwise results indicated significant size variation between *G. t. kalaharicus* and *G. t. punctatus* (R² = 0.116, F = 5.515, *P* = 0.021), *G. t. kalaharicus* and *G. deplanatus* (R² = 0.677, F = 110.907, *P* = 0.001), and *G. t. punctatus* and *G. deplanatus* (R² = 0.602, F = 86.106, *P* = 0.001) (Fig. 3A, B).

### Morphological variation among the studied taxa

Principal Component Analysis (PCA) of prothoracic shape variation showed that the first three principal components accounted for a cumulative variance of 44%, with PC1 explaining 17.9%, PC2 contributing 14%, and PC3 capturing 12.1% of the total shape variation (Fig. 3C). Along PC1, negative values were associated with a more compact and flattened morphology and development of the prosternal process. In contrast, positive values corresponded to a more pronounced and extended shape with a curved prosternal process (Fig. 3C). Along PC2, negative values were linked to a narrower pronotal width and a more tapered prosternal process. In contrast, positive values corresponded to a broader pronotal width and a more robust prosternal process structure (Fig. 3C). PC2 and PC3 provided additional variation in shape.

For the pterothorax, PCA indicated that the first three principal components accounted for a cumulative variance of 67.7%, with PC1 explaining 38.2%, PC2 contributing 15.9%, and PC3 capturing 13.6% of the total shape variation (Fig. 3D). Along PC1, negative values were linked to a more elongated and compressed morphology. In contrast, positive values corresponded to a broader, more rounded shape. PC2 and PC3 contributed further variation in shape (Fig. 3D).

### Allometry

Procrustes ANOVA for the prothorax revealed a statistically significant but weak allometric effect (R² = 0.044, F = 3.571, *P* = 0.002), with size (centroid size, CS) explaining 4.4% of the shape variation. For the pterothorax, a more substantial allometric effect was observed (R² = 0.184, F = 17.323, *P* = 0.001), with size accounting for 18.4% of the shape variation.

**Fig. 4:**
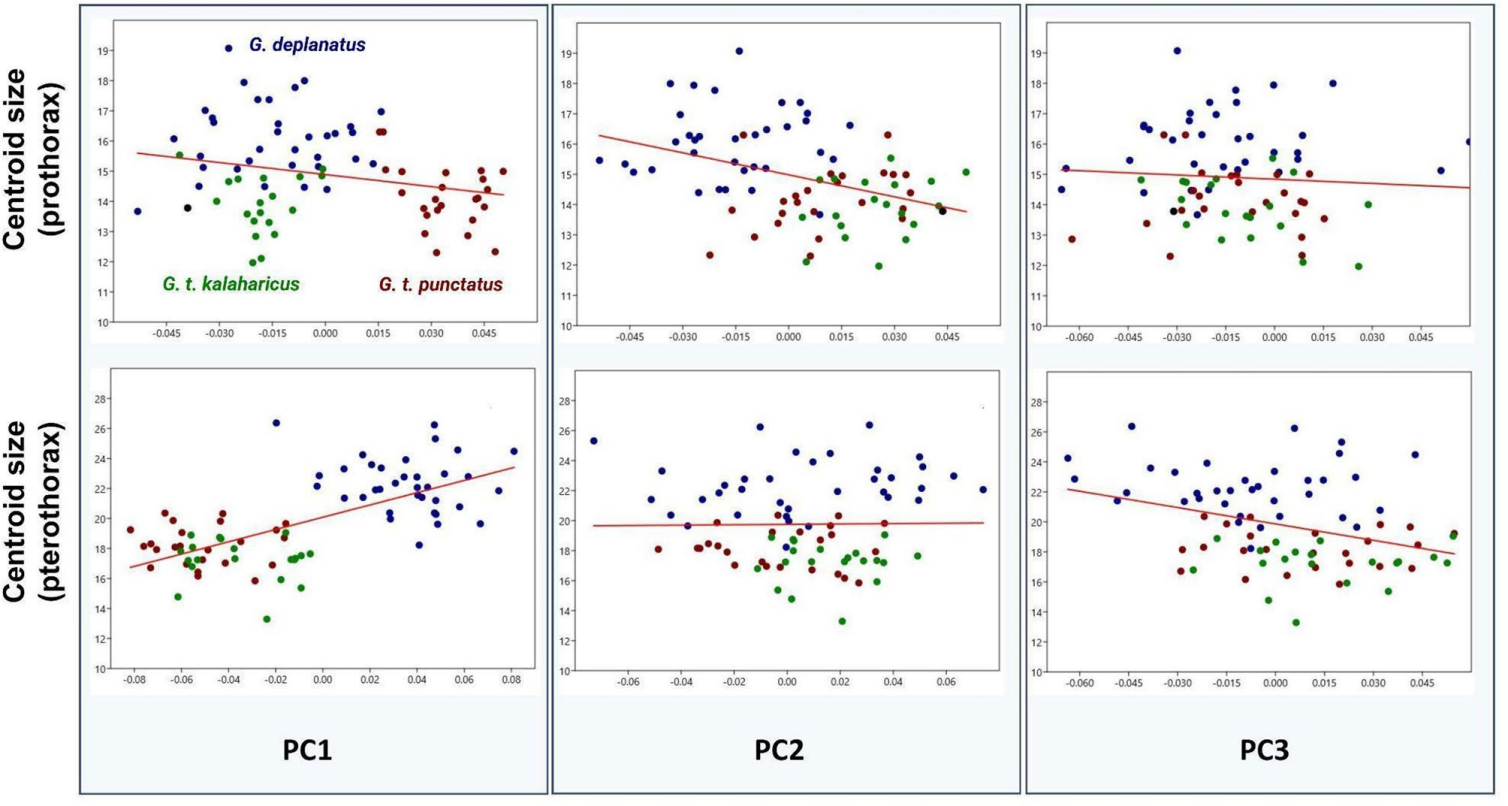
Relationships between prothoracic and pterothoracic centroid sizes and the first three principal components (PC1, PC2, PC3).

Figure 4 illustrates the relationships between prothoracic and pterothoracic centroid sizes and the first three principal components (PC1, PC2, PC3). No significant relationship was found between size and PC1 or PC3 for the prothorax, but PC2 showed a significant dependence on size (*P* < 0.001). For the pterothorax, significant relationships were observed between size and PC1 (*P* < 0.001) and PC3 (*P* < 0.05), while no significant relationship was detected with PC2 (Fig. 4). Shape models along these axes are depicted in Fig. 3D.

## Discussion

Traditional linear morphometric approaches have been widely used in entomology to quantify morphological differences among taxa^25,28,38–40^. While such linear analyses can provide valuable insights, they fail to capture the full extent of shape variation, particularly in structures where curvature, spatial relationships, and subtle deformations are crucial in distinguishing taxa. In contrast, geometric morphometrics offers a more comprehensive approach by analyzing shapes in a three-dimensional context (Figs. 3 and 4), preserving spatial relationships between anatomical landmarks^29,30,41^. This method enables a more detailed assessment of morphological differences and provides a robust framework for evaluating taxonomic distinctions that may be overlooked using traditional linear metrics. Despite such advantages this methodology has been largely neglected and remains untested for entomological research. In order to explore the applicability of 3D geometric morphometrics in systematic entomology, present study targeted three closely related taxa of sandworm beetles (i.e., *Gonopus* *deplanatus*, *G. t. kalaharicus*, *G. t. punctatus*) (Fig. 1).

The landmarks used in this study were carefully selected to correspond to anatomically distinct and repeatable structures, ensuring both the accuracy and reliability of the geometric morphometric approach^3,35^. By focusing on well-defined anatomical regions of the prothorax and pterothorax, this methodology minimizes subjectivity in landmark placement and enhances the consistency of shape data across specimens (Fig. 2). The reproducibility of these landmarks is crucial for taxonomic studies, as it allows for direct comparisons between different research efforts and ensures methodological continuity^42,43^. Furthermore, the standardized landmark configuration applied in this study provides a robust methodological reference for future investigations in entomology, particularly for other taxa within Tenebrionidae or even broader beetle lineages^34,44^. By demonstrating that 3D geometric morphometric analyses can be systematically applied to insect morphology, this study lays the groundwork for expanding its use in taxonomic revisions, evolutionary studies, and functional morphology assessments. The methodological framework presented here can serve as a guideline for researchers aiming to integrate quantitative shape analysis into systematic entomology, promoting the broader adoption of geometric morphometric techniques in the field.

The morphological differences observed in this study are significant for taxonomic classification and provide valuable insights into the ecological and functional adaptations of sandworm beetles^20^. While G. deplanatus displays coarse and easily definable morphological differences from the other taxa (Fig. 3B), our findings provide compelling evidence for differentiation of *G. t. kalaharicus* and *G. t. punctatus*. Namely, the latter taxon is characterised by a narrower pronotum, a more pronounced prosternal process (Fig. 3C), and a more elongate pterothorax (Fig. 3D). This result is particularly important, as previous classifications relied on subtle diagnostic features often considered insufficient for clear taxonomic separation20. Additionally, the variations between those subspecies suggest potential adaptive responses to different desert environments, highlighting the role of morphological traits in survival strategies. Given that *G. t. kalaharicus* and *G. t. punctatus* inhabit distinct desert ecosystems—Kalahari and Namib, respectively (Fig. 1C)—environmental pressures such as substrate composition, temperature fluctuations, and burrowing behavior could have driven these structural differences^1,45–47^. For instance, a more robust prothorax might enhance digging efficiency or provide increased protection against predation. At the same time, variations in pterothorax shape could be linked to locomotion efficiency in different sand types. This aligns with patterns observed in other desert-adapted Coleoptera, such as *Onymacris* Allard (Tenebrionidae) and *Carabus* Linnaeus (Carabidae), where pronotal and elytral shapes correlate with habitat-specific adaptations^48,49^. Future studies integrating behavioral data of sandworm beetles and environmental modeling could test the hypotheses above, linking shape differences to specific fitness advantages and evolutionary trajectories in desert ecosystems. A similar case can be drawn for *G. deplanatus*, which is characterised by uniquely widened pterothorax giving it a more rounded overall shape when compared to other studies’ taxa (Fig. 3D). Furthermore, as revealed here, larger individuals tend to have more pronounced and rounded elytra (Fig. 4). The omnipresence of *G. deplanatus* in different South African ecosystems (Fig. 1C) can suggest that its pterothoracic morphology can be potentially tied with thermoregulation, as the larger subelytral cavity provides a more efficient cooling system^50^. Despite its potentially fundamental importance, this phenomenon received little attention from researchers^51^. Nevertheless, as revealed here, the application of 3D geometric morphometrics can play a game-changing role as it enables quantification of traditionally indistinguishable properties of insect bodies.

While this study demonstrates the utility of 3D geometric morphometrics in systematic entomology, several limitations should be acknowledged. First, while landmark-based geometric morphometrics provides a robust framework for quantifying morphological differences, it is inherently limited by selecting landmarks restricted to homologous and easily identifiable anatomical points. This may exclude other subtle yet biologically relevant shape variations that could be captured through alternative approaches such as semi-landmark or outline-based analyses. Second, as most described insect species are exclusively known from relatively penurious type series or single specimens^8,49,52^, another limitation for 3D geometric morphometric studies in the context of taxonomy is the material availability. Relatively small sample sizes, which, while sufficient for detecting significant shape differences, may not fully represent the extent of morphological variation within each taxon. Moreover, the overwhelming lack of ecological and behavioral data for the majority precludes the direct correlation of morphological differences with functional adaptations, which limits the interpretation of taxonomic differentiation. Therefore, future studies should combine morphometric, molecular, and ecological approaches to understand a given biological problem comprehensively. Another technical challenge encountered in this study was the relatively small size of the specimens (13–24 mm), which complicated the scanning process. The delicate structure of beetle legs and antennae, in particular, posed difficulties during digitization, as their fine and thin morphology made it challenging to achieve a complete and accurate 3D reconstruction. To overcome this obstacle, the present study targeted large, heavily sclerotized portions of insects’ bodies (i.e., pro- and pterothorax). These challenges increased the complexity of data collection and required additional manual adjustments and repetitive scanning to ensure the quality of the final models (Fig. 3C, D). However, despite these difficulties, 3D geometric morphometric scanning provided a cost-effective and rapid alternative to high-resolution techniques such as microcomputed tomography (micro-CT). While micro-CT offers exceptional detail^14,15,50,53^, it requires specialized equipment and significantly longer processing times, precluding data collection on a large population scale^50^. In contrast, the method used in this study offers a more accessible and scalable approach for entomological research, making it a viable option for large-scale taxonomic studies where efficiency and affordability are key considerations.

## Conclusion

This study highlights the transformative potential of 3D geometric morphometrics in entomological research by providing a quantitative framework for distinguishing even closely related taxa. As revealed here, this methodology could have an especially significant impact on insect taxonomy, ecological and evolutionary studies. Despite challenges related to specimen size, landmark selection, and behavioral data availability, this study establishes 3D geometric morphometrics as a robust, accessible, and scalable tool for entomology. The observed morphological differences among studied sandworm beetle taxa not only support their taxonomic distinctiveness through previously overlooked traits but also suggest functional adaptations to distinct desert habitats, underscoring the link between form and environmental pressures.

## Electronic supplementary material

Below is the link to the electronic supplementary material.

Supplementary Material 1

## Data Availability

Data is provided within the manuscript or supplementary information files.

## References

[1] Koch, C. Composition, xerophilous character, and origin of the Namib Tenebrionidae. *Sci. Pap Namib Desert Res. Stn.* **5**, 66–69 (1962).

[2] Thomas, D. B. Patterns in the abundance of some tenebrionid beetles in the Mojave desert. *Environ. Entomol.* **8**, 568–574 (1979).

[3] Matthews, E. G., Lawrence, J. F., Bouchard, P., Steiner, W. E. & Ślipiński, S. A. *Tenebrionidae Latreille, 1802*. In *Handbook of Zoology. A Natural History of the Phyla of the Animal Kingdom* (eds Leschen, R. A. B., Beutel, R. G. & Lawrence, J. F.) 574–659 (Walter de Gruyter, 2010).

[4] Mitteroecker, P. & Gunz, P. Advances in geometric morphometrics. *Evol. Biol.* **36**, 235–247 (2010).

[5] Kergoat, G. J. et al. Higher-level molecular phylogeny of darkling beetles (Coleoptera: Tenebrionidae). *Syst. Entomol.* **39**, 486–499 (2014).

[6] Kergoat, G. et al. Cretaceous environmental changes led to high extinction rates in a hyperdiverse beetle family. *BMC Evol. Biol.* **14**, 220 (2014). 25331733 10.1186/s12862-014-0220-1 PMC4210489

[7] Wang, Y. et al. Adaptation of the egg of the desert beetle, *Microdera punctipennis* (Coleoptera: Tenebrionidae), to an arid environment. *J. Insect Sci.* **14**, 246 (2014). 25525108 10.1093/jisesa/ieu108 PMC5634134

[8] Kamiński, M. J. Catalogue and distribution of the subtribe Eurynotina (Coleoptera: tenebrionidae: Pedinini). *Ann. Zool.* **66**, 227–266 (2016).

[9] Bouchard, P. et al. Review of genus-group names in the family Tenebrionidae (Insecta, Coleoptera). *ZooKeys* **1050**, 1–633 (2021). 34385881 10.3897/zookeys.1050.64217 PMC8328949

[10] Johnston, M. A. et al. Testing the taxonomy of *Amphidorini* leconte (Coleoptera: Tenebrionidae): A molecular phylogeny leveraging museum sequencing. *Ann. Zool.* **72**, 49–68 (2022).

[11] Pinto, P. & Larrea-Meza, S. Zúñiga-Reinoso, Á. A new species of *Diastoleus* Solier (Coleoptera: Tenebrionidae) from the hyperarid Andes of the Atacama desert of Chile. *Ann. Zool.* **72**, 91–96 (2022).

[12] Silvestro, V. A., Macagno, H. B. & Flores, G. E. Definition of the *Emmallodera perlifera* species group (Coleoptera: tenebrionidae: Scotobiini) from Argentina with descriptions of two new species. *Ann. Zool.* **72**, 81–90 (2022).

[13] Ragionieri, L., Zúñiga-Reinoso, Á., Bläser, M. & Predel, R. Phylogenomics of darkling beetles (Coleoptera: Tenebrionidae) from the Atacama desert. *PeerJ* **11**, e14848 (2023). 36855434 10.7717/peerj.14848 PMC9968461

[14] Raś, M., Kamiński, M. J. & Iwan, D. Fossoriality in desert-adapted tenebrionid (Coleoptera) larvae. *Sci. Rep.* **12**, 13233 (2022). 35918527 10.1038/s41598-022-17581-6 PMC9346125

[15] Raś, M., Wipfler, B., Dannenfeld, T. & Iwan, D. Postembryonic development of the tracheal system of beetles in the context of aptery and adaptations towards an arid environment. *PeerJ* **10**, e13378 (2022). 35855904 10.7717/peerj.13378 PMC9288169

[16] Cheli, G. H., Bosco, T. & Flores, G. The role of *Nyctelia dorsata* fairmaire, 1905 (Coleoptera: Tenebrionidae) on litter fragmentation processes and soil biogeochemical cycles in arid patagonia. *Ann. Zool.* **72**, 129–134 (2022).

[17] Nørgaard, T. & Dacke, M. Fog-basking behaviour and water collection efficiency in Namib desert darkling beetles. *Front. Zool.* **7**, 23 (2010). 20637085 10.1186/1742-9994-7-23 PMC2918599

[18] Comanns, P. Passive water collection with the integument: mechanisms and their biomimetic potential. *J. Exp. Biol.* **221**, jeb153130 (2018). 29789349 10.1242/jeb.153130

[19] Burrell, A. L., Evans, J. P. & De Kauwe, M. G. Anthropogenic climate change has driven over 5 million km² of drylands towards desertification. *Nat. Commun.* **11**, 3853 (2020). 32737311 10.1038/s41467-020-17710-7 PMC7395722

[20] Endrödy-Younga, S. A revision of the subtribe gonopina (Coleoptera: tenebrionidae: opatrinae: Platynotini). *Ann. Transvaal Mus.* **37**, 1–54 (2000).

[21] Iwan, D. Generic classification of the tribe Platynotini (Coleoptera: Tenebrionidae), with notes on phylogeny. *Ann. Zool.* **52**, 1–149 (2002).

[22] Rasa, O. A. E. Parabiosis and its proximate mechanisms in four Kalahari desert tenebrionid beetles. *Ethology* **98**, 137–148 (1994).

[23] Rasa, O. A. E. To stay or to leave? Decision rules for partner species relocation in two symbiotic pairs of desert beetles. *Anim. Cogn.* **1**, 47–54 (1998).

[24] Crawford, C. S. The community ecology of macroarthropod detritivores. In The Ecology of Desert Communities (ed Polis, G. A.) 89–112 (Univ. of Arizona, (1991).

[25] Tatsuta, H., Takahashi, K. H. & Sakamaki, Y. Geometric morphometrics in entomology: basics and applications. *Entomol. Sci.* **21**, 164–184 (2018).

[26] Romiti, F., DeZan, L. R. & Piras, P. Shape variation of mandible and head in *Lucanus cervus* (Coleoptera: Lucanidae): A comparison of morphometric approaches. *Biol. J. Linn. Soc.* **120**, 1–16 (2016).

[27] Leonardi, M. S. et al. The deeper the rounder: body shape variation in lice parasitizing diving hosts. *Sci. Rep.* **14**, 20947 (2024). 39251772 10.1038/s41598-024-71541-w PMC11385217

[28] Zúñiga-Reinoso, Á. & Benítez, H. A. The overrated use of the morphological cryptic species concept: an example with *Nyctelia* dark beetles (Coleoptera: Tenebrionidae) using geometric morphometrics. *Zool. A*. **255**, 47–53 (2015).

[29] Fabre, P. H., Hautier, L., Dimitrov, D. & Douzery, E. J. P. A glimpse on the pattern and timing of diversification in the genus *Apodemus* (Rodentia: Muridae). *Biol. J. Linn. Soc.* **111**, 487–508 (2014).

[30] Ilić, M., Tomasović, G., Miličić, D. & Urošević, A. Morphometric analysis of *Lucanus cervus* populations in Europe. *J. Morphol.* **280**, 1367–1378 (2019).

[31] Klingenberg, C. P. Size, shape, and form: concepts of allometry in geometric morphometrics. *Dev. Genes Evol.* **226**, 113–137 (2016). 27038023 10.1007/s00427-016-0539-2 PMC4896994

[32] Gündemir, O. & Szara, T. Morphological patterns of the European bison (*Bison bonasus*) skull. *Sci. Rep.* **15**, 1418 (2025). 39789289 10.1038/s41598-025-85654-3 PMC11718087

[33] Batur, B. et al. Geometric morphometric analysis of plastinated brain sections using computer-based methods: evaluating shrinkage and shape changes. *Ann. Anat.* **257**, 152351 (2025). 39547470 10.1016/j.aanat.2024.152351

[34] Doyen, J. T. & Tschinkel, W. R. Phenetic and cladistic relationships among tenebrionid beetles (Coleoptera). *Syst. Entomol.* **7**, 127–183 (1982).

[35] Doyen, J. T. The skeletal anatomy of *Tenebrio molitor* (Coleoptera: Tenebrionidae). *Misc Publ Entomol. Soc. Am.* **5**, 103–150 (1966).

[36] Rolfe, S. et al. SlicerMorph: an open and extensible platform to retrieve, visualize and analyse 3D morphology. *Methods Ecol. Evol.* **12**, 1816–1825 (2021). 40401087 10.1111/2041-210x.13669 PMC12094517

[37] Adams, D. C. & Otárola-Castillo, E. Geomorph: an R package for the collection and analysis of geometric morphometric shape data. *Methods Ecol. Evol.* **4**, 393–399 (2013).

[38] Sauer, F. G., Jaworski, L. & Erdbeer, L. Geometric morphometric wing analysis represents a robust tool to identify female mosquitoes (Diptera: Culicidae) in Germany. *Sci. Rep.* **10**, 17613 (2020). 33077803 10.1038/s41598-020-72873-z PMC7573584

[39] Zhang, M. et al. Geometric morphometric analysis of the pronotum and elytron in stag beetles: insight into its diversity and evolution. *ZooKeys* **833**, 21 (2019). 31015774 10.3897/zookeys.833.26164 PMC6443621

[40] Taravati, S., Darvish, J. & Mirshamsi, O. Geometric morphometric study of two species of the psammophilous genus erodiontes (Coleoptera: Tenebrionidae) from the lute desert, central Iran. *Iran. J. Anim. Biosystematics*. **5** (2), 81–89 (2009).

[41] Zelditch, M., Swiderski, D. & Sheets, H. D. *Geometric Morphometrics for Biologists: A Primer* (Academic, 2012).

[42] Toma, A. M. et al. Reproducibility of facial soft tissue landmarks on 3D laser-scanned facial images. *Orthod. Craniofac. Res.* **12**, 33–42 (2009). 19154273 10.1111/j.1601-6343.2008.01435.x

[43] Corte-Real, A., Kato, R. M., Nunes, T., Vale, F. & Garib, D. Reproducibility of mandibular landmarks for three-dimensional assessment. *Forensic Sci. Int. Rep.* **2**, 100144 (2020).

[44] Barclay, M. V. L. & Bouchard, P. *Beetles of the World: A Natural History* (Princeton University Press, 2023).

[45] Draney, M. L. The subelytral cavity of desert tenebrionids. *Fla. Entomol.* **76**, 539–549 (1993).

[46] Fattorini, S. Darkling beetle communities in two geologically contrasting biotopes: testing biodiversity patterns by microsite comparisons. *Biol. J. Linn. Soc.* **98**, 787–793 (2009).

[47] Fattorini, S. Adaptations of tenebrionid beetles to mediterranean sand Dune environments and the impact of climate change (Coleoptera: Tenebrionidae). *Fragmenta Entomol.* **55**, 1–20 (2023).

[48] Lamb, T. & Bond, J. E. A multilocus perspective on phylogenetic relationships in the Namib darkling beetle genus *Onymacris* (Tenebrionidae). *Mol. Phylogenet. Evol.* **66** (3), 757–765 (2013). 23159892 10.1016/j.ympev.2012.10.026

[49] Alibert, P., Moureau, B., Dommergues, J. L. & David, B. Differentiation at a microgeographical scale within two species of ground beetle, Carabus auronitens and C. nemoralis (Coleoptera, arabidae): A geometrical morphometric approach. *Zool. Scr.* **30**, 299–311 (2001).

[50] Fiori, G. *La Cavita Sottoelitrale Dei Tenebrionidi Apomorfi*60 (Redia, 1977).

[51] Goczał, J. & Beutel, R. G. Beetle elytra: evolution, modifications and biological functions. *Biol. Lett.* **19** (3), 20220559 (2023). 36855857 10.1098/rsbl.2022.0559 PMC9975656

[52] Kamiński, M. J. et al. Reevaluation of *Blapimorpha* and *Opatrinae*: addressing a major phylogeny-classification gap in darkling beetles (Coleoptera: tenebrionidae: Blaptinae). *Syst. Entomol.* **46**, 140–156 (2021).

[53] Iwan, D., Kamiński, M. J. & Raś, M. The last breath: A µCT-based method for investigating the tracheal system in Hexapoda. *Arthropod Struct. Dev.* **44**, 218–227 (2015). 25791158 10.1016/j.asd.2015.02.002

