# Supplementary material for: Exploring desert-adapted beetles with 3D geometric morphometrics
Source: Sci Rep. 2025 Jul 2;15:22824. doi: 10.1038/s41598-025-05967-1 (PMC12214608; doi:10.1038/s41598-025-05967-1)
Supplement: Supplementary file 1 — Supplementary Material 1 [file 41598_2025_5967_MOESM1_ESM.docx]

Supplementary data for the paper entitled *Exploring Desert-Adapted Beetles with 3D Geometric Morphometrics: A New Approach to Systematic Entomology*.

**Appendix. *Label data and repositories for the studied Gonopus specimens.***

**I. *Gonopus* (*Gonopus*) *deplanatus* Fåhraeus**

**Naturhistorisches Museum Basel, Basel, Switzerland**: Specimen 1: "Coll. G. Frey / NMB", "Dr. Pöch, '07.09 / Süd-Afrika", "Ngamiland". Specimen 2: "Gonopus deplanatus Fahr. det H. Kulzer 1952", "Coll. G. Frey / NMB", "Dr. Pöch, '07.09 / Süd-Afrika". Specimen 3: "Gonopus agrestis Fahr.", "Coll. G. Frey / NMB". Specimen 4: "Coll. G. Frey / NMB", "Capland", "Gonopus agrestis Fahr.". Specimen 5: "Gobabis", "Coll. G. Frey / NMB", "Coll. G. Frey / NMB". Specimen 6: "Dr. Penther Süd-Africa", "Gonopus deplanatus Fahr. det H. Kulzer 1952", "Coll. G. Frey / NMB". Specimen 7: "Dr. Penther Süd-Africa", "Coll. G. Frey / NMB". Specimen 8: "Dr. Penther Süd-Africa", "Coll. G. Frey / NMB". Specimen 9: "Tsumeb. S.W. Afr. leg. Grany, XII 1964", "Coll. G. Frey / NMB". Specimen 10: "Angola Miss. sci. Suisse 1928-29 ", "Gonopus deplanatus Fahr.", "Coll. G. Frey NMB", "angola XI.". Specimen 11: "Tsumeb, S.W. Afr. leg. Garny, XII.1964", "Coll. G. Frey NMB". Specimen 12: "Ukuanyama", "Angola W. Hüber lgt.", "Coll. G. Frey NMB". Specimen 13: "Gobabis", "Coll. G. Frey / NMB", "Coll. G. Frey / NMB". Specimen 14: "Windhoek", "Coll. G. Frey NMB", "Gonopus deplanatus Fahr.", "ex Coll. Bennigsen". Specimen 15: "Tsumeb, S.W. Afr. leg. Garny, XII.1964", "Coll. G. Frey NMB". Specimen 16: "Tsumeb, S.W. Afr. leg. Garny, 1964", "Coll. G. Frey NMB". Specimen 17: "Gobabis", "Coll. G. Frey NMB", "Gonopus deplanatus Fahr.". Specimen 18: "Windhoek", "Coll. G. Frey NMB", "Gonopus deplanatus Fahr.", "ex Coll. Bennigsen". Specimen 19: "Tsumeb, S.W. Afr. leg. Garny, 1964", "Coll. G. Frey NMB".

**Museum and Institute of Zoology, Polish Academy of Sciences, Warszawa, Poland**: Specimen 20: "Namibia Kuzikus Camp 23°13'48'' S 18°24'02''E leg. D. Schimrosczyk". Specimen 21: "Namibia Kuzikus Camp 23°13'48'' S 18°24'02''E leg. D. Schimrosczyk". Specimen 22: "Namibia Kuzikus Camp 23°13'48'' S 18°24'02''E leg. D. Schimrosczyk". Specimen 23: "Namibia Kuzikus Camp 23°13'48'' S 18°24'02''E leg. D. Schimrosczyk". Specimen 24: "ex coll. Witold Eichler Inst. Zool PAN Warszawa 64/63", "N Rhodesia 3 XII 1943 Dr W. Eichler". Specimen 25: "Namibia Kuzikus Lodge 23°14'15'' S 18°23'23''E leg. J. Reinhard", "09.V.2011". Specimen 26: "ex coll. Witold Eichler Inst. Zool PAN Warszawa 64/63", "N Rhodesia I 1944 Dr W. Eichler". Specimen 27: "181", "2011.IV.14". Specimen 28: "Gonopus agrestis", "Mus. Zool. Polon. Warszawa 36/5".

**Museum für Naturkunde, Berlin, Germany**: Specimen 29: "85046", "D. S. W. Afrika Omahene IX-X 04, L. Trotha S.", "GONOPUS agristes Fahr. det. Endrödy-Younga", "BERLIN". Specimen 30: "85046", "D. S. W. Afrika Omahene IX-X 04, L. Trotha S.", "GONOPUS agristes Fahr. det. Endrödy-Younga", "BERLIN".

**The Natural History Museum, London, United Kingdom**: Specimen 31: "Pascoe Coll. 93-60", "Brithish Museum".

**South African National Collection of Insects; Pretoria, South Africa**: Specimen 32: "SOUTH AFRICA: LIMPOPO Trap15-5, Malebogo NR nr. Blouberg 23°17’01S 28°50’15E 13-22.xii. 2006, M. Burger, B. Chamberlain, R. Hawkins & D. Maguire", "NATIONAL COLL OF INSECTS Pretoria, South Africa", "Caught in pitfall and funnel-traps Trap 15-5". Specimen 33: "181", "2011.IV.14". Specimen 34: "SOUTH AFRICA: LIMPOPO Trap15-5, Malebogo NR nr. Blouberg 23°17’01S 28°50’15E 13-22.xii. 2006, M. Burger, B. Chamberlain, R. Hawkins & D. Maguire", "NATIONAL COLL OF INSECTS Pretoria, South Africa", "Caught in pitfall and funnel-traps Trap 15-5". Specimen 35: "SOUTH AFRICA: LIMPOPO Trap15-5, Malebogo NR nr. Blouberg 23°17’01S 28°50’15E 13-22.xii. 2006, M. Burger, B. Chamberlain, R. Hawkins & D. Maguire", "NATIONAL COLL OF INSECTS Pretoria, South Africa", "Caught in pitfall and funnel-traps Trap 15-5".

**II. *Gonopus* (*Gonopus*) *tibialis kalaharicus* Endrödy-Younga**

**Natural History Museum of Erfurt, Germany**: Specimen 1: “SüdAfrika, C.P. N Upington, km 208 R 360 10.03.1994 Arndt & Gröger”. Specimen 2: “SüdAfrika, C.P. N Upington, km 208 R 360 10.03.1994 Arndt & Gröger”. Specimen 3: “SüdAfrika, C.P. N Upington, km 208 R 360 10.03.1994 Arndt & Gröger”. Specimen 4: “SüdAfrika, C.P. Kalahari Mata-Mata 10.-12.03.1994 Arndt & Gröger”. Specimen 5: “SüdAfrika, C.P. N Upington, km 208 R 360 10.03.1994 Arndt & Gröger”. Specimen 6: “SüdAfrika, C.P. N Upington, km 208 R 360 10.03.1994 Arndt & Gröger”. Specimen 7: “SüdAfrika, C.P. Kalahari Mata-Mata 10.-12.03.1994 Arndt & Gröger”. Specimen 8: “Namibia 8.II.2006 Maltahohe Helmeringhausen Werner & Smrz leg”. Specimen 9: “SüdAfrika, C.P. N Upington, km 208 R 360 10.03.1994 Arndt & Gröger”. Specimen 10: “Namibia 8.II.2006 Maltahohe Helmeringhausen Werner & Smrz leg”. Specimen 11: “SüdAfrika, C.P. Kalahari Mata-Mata 10.-12.03.1994 Arndt & Gröger”.

**Museum für Naturkunde, Berlin, Germany**: Specimen 12: “Namibia 8.II.2006 Maltahohe Helmeringhausen Werner & Smrz leg”. Specimen 13: “Namibia 8.II.2006 Maltahohe Helmeringhausen Werner & Smrz leg”. Specimen 14: “Namibia 8.II.2006 Maltahohe Helmeringhausen Werner & Smrz leg”. Specimen 15: “Namibia 8.II.2006 Maltahohe Helmeringhausen Werner & Smrz leg”. Specimen 16: “Namibia 8.II.2006 Maltahohe Helmeringhausen Werner & Smrz leg”.

**Ditsong National Museum of Natural History, Pretoria, South Africa**: Specimen 17: “Swedish South Africa Expedition 1950-1951 Brinck - Rudebeck”, “S. Afr. Cape Prov. Tweede Rivieren, Kalahari Gemsbok Park 16-18.XI.50 No. 53”. Specimen 18: “Swedish South Africa Expedition 1950-1951 Brinck - Rudebeck”, “S. Afr. Cape Prov. Tweede Rivieren, Kalahari Gemsbok Park 16-18.XI.50 No. 53”. Specimen 19: “Swedish South Africa Expedition 1950-1951 Brinck - Rudebeck”, “S. Afr. Cape Prov. Tweede Rivieren, Kalahari Gemsbok Park 16-18.XI.50 No. 53”. Specimen 20: “Swedish South Africa Expedition 1950-1951 Brinck - Rudebeck”, “S. Afr. Cape Prov. Tweede Rivieren, Kalahari Gemsbok Park 16-18.XI.50 No. 53”.

**III. *Gonopus* (*Gonopus*) *tibialis punctatus* Endrödy-Younga**

**The Natural History Museum, London, United Kingdom:**

Specimen 1: "S.W. Africa Sesriem Farm Maltahoe distr. 19-20 i. 1972". Specimen 2: "Nevinson Coll. 1916-14". Specimen 3: "Nevinson Coll. 1916-14". Specimen 4: "S.W. Africa Sesriem Farm Maltahoe distr. 19-20 i. 1972". Specimen 5: "S.W. Africa Sesriem Farm Maltahoe distr. 19-20 i. 1972". Specimen 6: "S.W. Africa Sesriem Farm Maltahoe distr. 19-20 i. 1972". Specimen 7: "Nevinson Coll. 1916-14".

Specimen 8: "S.W. Africa Sesriem Farm Maltahoe distr. 19-20 i. 1972". Specimen 9: "S.W. Africa Sesriem Farm Maltahoe distr. 19-20 i. 1972". Specimen 10: "S.W. Africa Sesriem Farm Maltahoe distr. 19-20 i. 1972".

**Museum für Naturkunde, Berlin, Germany:**

Specimen 11: "Namibia 13.-15.III.1999 24 33'S/15 46'E Namib Naukluft Park: Sesriem leg. B. + M. Uhlig". Specimen 12: "Namibia 13.-15.III.1999 24 33'S/15 46'E Namib Naukluft Park: Sesriem leg. B. + M. Uhlig". Specimen 13: "Namibia 13.-15.III.1999 24 33'S/15 46'E Namib Naukluft Park: Sesriem leg. B. + M. Uhlig". Specimen 14: "Namibia 13.-15.III.1999 24 33'S/15 46'E Namib Naukluft Park: Sesriem leg. B. + M. Uhlig".

**Ditsong National Museum of Natural History, Pretoria, South Africa:**

Specimen 15: "Sossusvlei J. Boomker", "10.4.1977 S.W.A.". Specimen 16: "Sossusvlei J. Boomker", "10.4.1977 S.W.A.". Specimen 17: "Sossusvlei J. Boomker", "10.4.1977 S.W.A.". Specimen 18: "S.W. Afr. Namib farm Kanaan 25.53S-16.07E", "7.5.1977; E-Y: 1323 singled on dunes Steven Mothlasedi".

Specimen 19: "Sossusvlei VII.1982 E. Holm". Specimen 20: "Sossusvlei J. Boomker", "10.4.1977 S.W.A.". Specimen 21: "Sossusvlei J. Boomker", "10.4.1977 S.W.A.". Specimen 22: "F. Bates 81-19", "Gonopus sulcatus var. Sol". Specimen 23: "Gonopus exaratus lec. Gehin". Specimen 24: "Sossusvlei VII.1982 E. Holm".
